# Associating liver partition and portal vein ligation for staged hepatectomy in Qatar: Initial experience with two case series and review of the literature

**DOI:** 10.1016/j.ijscr.2019.03.060

**Published:** 2019-04-06

**Authors:** Ibnouf Sulieman, Walid Elmoghazy, Mohammed Said Ghali, Ahmed Mahfouz, Ahmed Elaffandi, Hatem Khalaf

**Affiliations:** a Department of Surgery, Division of Organ Transplant, Hamad General Hospital, Doha, PO Box 3050, Qatar; b Department of Surgery, Sohag University, Sohag, Egypt; c Department of Surgery, Ain Shams University, Cairo, Egypt; d Department of Radiology, Hamad General Hospital, Doha, PO Box 3050, Qatar; e Department of Surgical Oncology, National Cancer Institute, Cairo University, Egypt

**Keywords:** ALPPS, associating liver partition and portal vein ligation for staged hepatectomy, FLR, future liver remnant, CT, computed tomography, MRI, magnetic resonance imaging, FDG-PET, fluorodeoxyglucose positron emission tomography, CUSA, cavitron ultrasonic surgical aspirator, ALT, alanine transaminase, AST, aspartate transaminase, IVC, inferior vena cava, RFA, radiofrequency ablation, PVL, portal vein ligation, PVE, portal vein embolizainferior vena cavation, ALTPS, associating liver tourniquet and portal vein occlusion for staged hepatectomy, RALPP, radiofrequency assisted liver partition, SPECT, single photon emission computed tomography, Liver, Resection, Associating liver partition and portal vein ligation for staged hepatectomy, ALPPS, Surgery

## Abstract

**Introduction:**

Associating Liver Partition and Portal Vein Ligation for Staged -hepatectomy (ALPPS) is an evolving procedure that allows rapid hypertrophy of the future liver remnant (FLR). We describe the first two cases performed in Qatar.

**Case presentations:**

**Case 1:** A 53 -year old male with sarcoma metastases to the liver 8 years after resection of an abdominal wall tumor, requiring an extended right hepatectomy but with in an inadequate FLR. ALPPS was done and he achieved 147% increase in the volume of the FLR within 6 days (from 15.9%–34.2%). The second stage was completed successfully on day 7. **Case 2:** A 59-year old male patient had colorectal liver metastases that required an extended right liver resection and had inadequate FLR of 19.8%. Seven days after the first stage, the FLR hypertrophied to 37.7% (90.2% increase in volume) and the second stage was completed successfully on day 8. Both patients had uneventful recovery and no recurrence or complications on follow up.

**Discussion:**

ALPPS allows large liver resections while circumventing the long delay in the conventional two staged hepatectomy and portal vein ligation/embolization. The reported morbidity and mortality in earlier series was high, but recent selection criteria and technique refinements reduce this morbidity. Many variations are still being reported.

**Conclusion:**

ALPPS is an evolving technique that adds to the armamentarium of the liver surgeon to allow larger liver resections in a timely manner. It is feasible and safe to be performed with careful selection.

## Highlights

• The volume of the liver that can be resected is limited by the need to leave behind liver tissue of sufficient volume to carry our normal liver functions and allow regeneration.
• Two staged hepatectomy and portal vein embolization/ligation allow hypertrophy of the future liver remnant, but the long period required may allow tumor progression.
• ALPPS combines portal vein ligation with transection of the liver tissue, inducing rapid hypertrophy of the future remnant liver.
• Despite high morbidity and mortality initially, the procedure is now safe due to better technique and the selection criteria.
• Future efforts are directed towards better evaluation of liver function, refinements in the technique, and more accurate patient selection.

## 1 Introduction

Liver resection is the only curative treatment for many primary neoplastic lesions. It is also indicated for liver metastases from many tumors, and the indications for resection of metastases is increasing with accumulating evidence of benefit in many tumors. The main limiting factor when evaluating these tumors is the ability to keep a liver volume after resection that will be adequate to support normal liver function and allow regeneration. In many instances, this is not possible, and the predicted future liver remnant will be judged to be too small, prohibiting resection. Several techniques have been devised to increase the volume of the FLR including portal vein ligation, portal vein embolization and two staged hepatectomy. These procedures increase the volume of the FLR and allow resection in many cases that would otherwise be unresectable. However, they are limited in the degree of hypertrophy they can induce, and the time required for such hypertrophy is extended, allowing progression in some tumors.

Associating liver partition and portal vein ligation (ALPPS) is a new procedure that combines the transection of liver parenchyma and the ligation of the portal vein to the diseased liver. It allows higher degrees of hypertrophy of the FLR in a short time, circumventing many of the shortcomings of the previous techniques and broadening the spectrum of the resectable liver lesions. The procedure was associated with high morbidity and mortality rates when first introduced, and the indications were not fully defined. Fortunately, refinements in the technique and the introduction of well-defined patient selection criteria resulted in improvements in the surgical outcomes and a better safety profile. The surgery is still evolving with new modifications being introduced on many fronts, aiming to improve the outcome further.

In this paper, we describe the first two case of ALPPS performed in Qatar and present a review of the literature encompassing the physiological basis, classical descriptions, modifications, and the future advances of this surgical procedure.

Both patients in this report were treated in Hamad General Hospital in Qatar. It is the main academic center in Qatar and is part of Hamad Medical Corporation. This manuscript was written following the PROCESS guidelines [1]

## 2 Methods

### 2.1 Ethical approval and registration

Ethical approval was obtained from the Hamad Medical Corporation (HMC) Medial Research Center (MRC) with the approval number MRC-04-18-107. This case series was also registered on the Research Registry [2] (UIN: researchregistry4672). Written informed consent was obtained from both patients for publication of this case series and the images and is available for review on request. All the procedures were in accordance with the ethical standards implemented by the HMC medical research center and the declaration of Helsinki.

### 2.2 Study design

This case series is a single center retrospective review of consecutive cases.

### 2.3 Setting

The cases were treated in Hamad General Hospital, which is an academic medical center and the main tertiary care center in Qatar. It is part of the Hamad Medical Corporation.

## 3 Patients

### 3.1 First patient

**Fig. 1:**
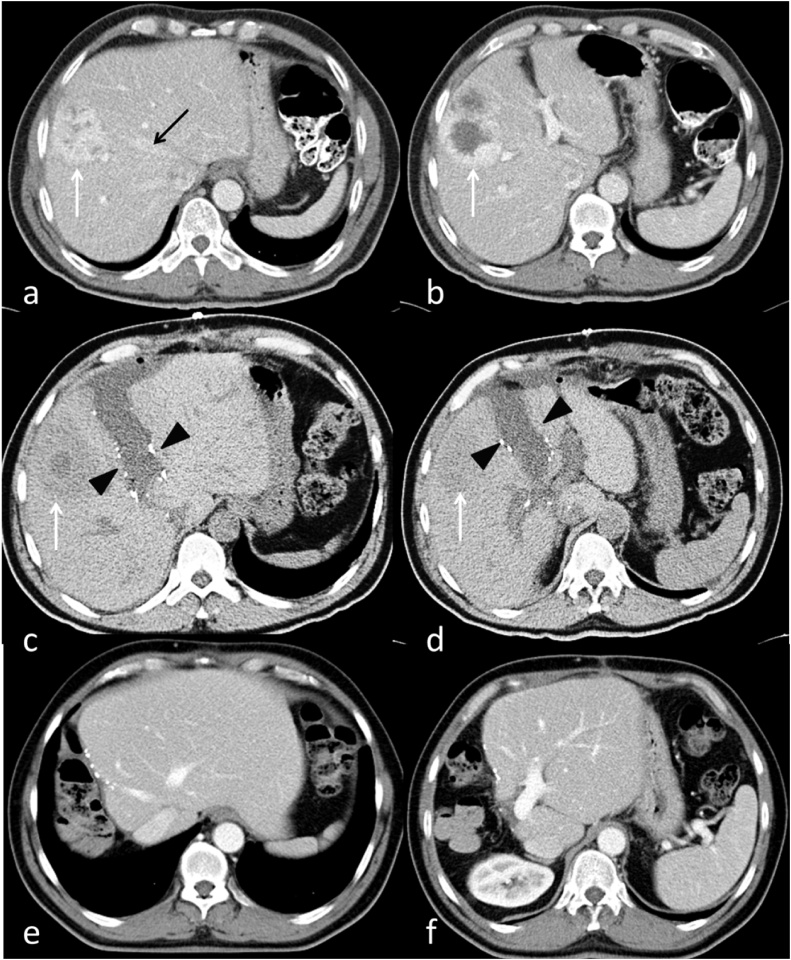
Patient 1: Preoperative contrast-enhanced CT (a,b) shows liver metastasis of the right lobe of the liver (white arrows), well encroaching on the plane of the middle hepatic vein (black arrow). Post-stage-1 unenhanced CT on postoperative day 7 (c,d) shows the liver partition plane metallic clips and postoperative fluid (black arrowheads) with normal liver parenchyma separating it from the lesion (white arrows). Post-stage-2 contrast-enhanced CT at 4 months post stage 2 (e,f) shows obvious increase in size of the remaining liver.

A 53-year old male patient was referred to our hospital after routine abdominal ultrasound for the investigation of hypertension. He was incidentally found to have 2 liver lesions, one lesion in segment VI measuring 34 × 37 mm in diameter, and the other one in segment IV measuring 60 × 46 mm in diameter. The patient had a history of excision of an abdominal tumor abroad 8 years prior to his presentation. It was reported as abdominal wall sarcomatous tumor; however, there was no detailed information about the origin or the histopathology of the tumor. A contrast-enhanced computed tomography (CT) scan and magnetic resonance imaging (MRI) confirmed presence of two liver lesions in segment VIII and segment VI, with only peripheral enhancement in the venous phase, but they could not characterize them (Fig. 1a and b). The lesions demonstrated increased uptake on fluorodeoxyglucose positron emission tomography (FDG-PET) images and no lesions were seen outside the liver. Ultrasound guided core needle biopsy revealed a malignant spindle cell neoplasm of at least grade 2. The case was discussed in the hepatobiliary multidisciplinary meeting and liver resection was decided based on the pathology and the indolent course with 8 years from the initial abdominal wall tumor resection. An extended right hepatectomy was required for adequate resection of the lesions.

CT volumetry revealed a total liver volume of 1802 ml and the calculated FLR after the proposed extended right hepatectomy was 287 ml (15.9%). We opted to perform ALPPS due to the unknown nature of the primary, lack of neo-adjuvant chemotherapy, and the small size of the FLR. We decided that the long waiting period of two-stage hepatectomy or portal vein embolization would be too risky considering the borderline resectability and the unclear nature of the tumor.

**Fig. 2:**
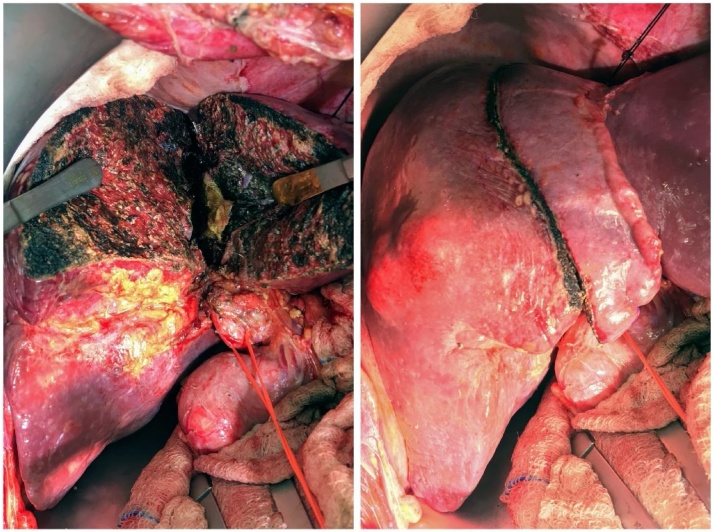
Patient 1: Intraoperative photograph of the liver after parenchymal division during the first stage. The right bile duct and the right hepatic artery are slung by vascular tape, while the right portal vein has been divided by a vascular stapler.

The patient underwent the first stage ALPPS, where the abdomen was accessed through an inverted L incision and adhesion from the prior surgery were identified and released. Dissection was carried out in the porta hepatis and all structures were identified, and the right hepatic lobe was fully mobilized. The right hepatic vein was identified and encircled, and the middle hepatic vein was preserved. Next, the right portal vein was divided using a vascular stapler. The line of dissection was identified 2 cm to the right of the falciform ligament, and parenchymal transection was carried out by Cavitron Ultrasonic Surgical Aspirator (CUSA^®^) [3] and Thunderbeat^®^ [4] (Fig. 2). The liver was found to be very vascular due to the metastatic involvement, and bleeding was encountered but controlled with clips and hemostatic agents. The operative time was 640 min and the blood loss was estimated to be 1000 ml, and the patient remained stable throughout the surgery. The patient recovered well after surgery and liver functions peaked at postoperative day 1 to a serum bilirubin of 27 umol/liter, alanine transaminase (ALT) of 254 U/L, and aspartate transaminase (AST) of 228 U/L, then started to decrease gradually and normalized by day 3.

CT volumetry was done on postoperative day 7 and showed an FLR volume of 705 ml and total liver volume of 2059 ml (Fig. 1c and d). This translated to an FLR of 34.2%. This comprises an increase of the FLR of 147% in 6 days. His liver function tests showed a total bilirubin level of 5 umol/liter, ALT 53 U/L, and AST 26 U/L.

**Fig. 3:**
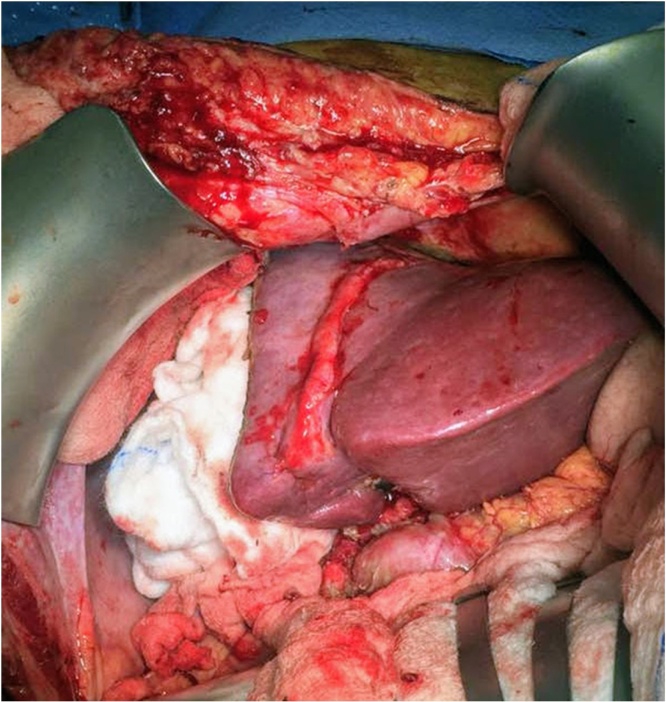
Patient 1: Intraoperative photograph of the liver after completion of the second stage. The disease right liver was resected, and the remnant left liver shows significant hypertrophy when compared to stage 1.

The patient underwent the second stage next day (day 8), where the abdomen was re-explored and the right hepatic artery, right bile duct, right hepatic vein and middle hepatic vein were divided, and the transected part of the liver was removed (Extended right hepatectomy). The operation lasted for about 2 h, with no blood loss (Fig. 3). The patient recovered uneventfully and remained stable apart from an episode of superficial thrombophlebitis, treated by antibiotics, and a small right subdiaphragmatic fluid collection that was treated conservatively. Serum bilirubin showed slight increase to 20umol/liter, serum ALT and AST peaked to 97 U/L and 46 U/L at day 1 and normalized by post-operative day 2.

The patient was discharged 5 days after the second stage. Histopathology showed high grade undifferentiated sarcoma with two main lesions, the largest 8 cm in size, and one small satellite lesion. The margins were clear with the closest margin to the transection line was 12 mm. The patient had adjuvant chemotherapy 4 weeks after his surgery with 6 cycles of Doxorubicin and ifosfamide over a 12-week period.

The patient had a follow up CT scan at 4 months (Fig. 1e and f) after surgery and a PET-CT scan at 6 months, and both demonstrated enormous regeneration of the liver remnant. Both images also showed no evidence of recurrence. On Follow-up at 18 months after surgery the patient remained asymptomatic, and MRI showed no evidence of recurrence.

### 3.2 Second patient

**Fig. 4:**
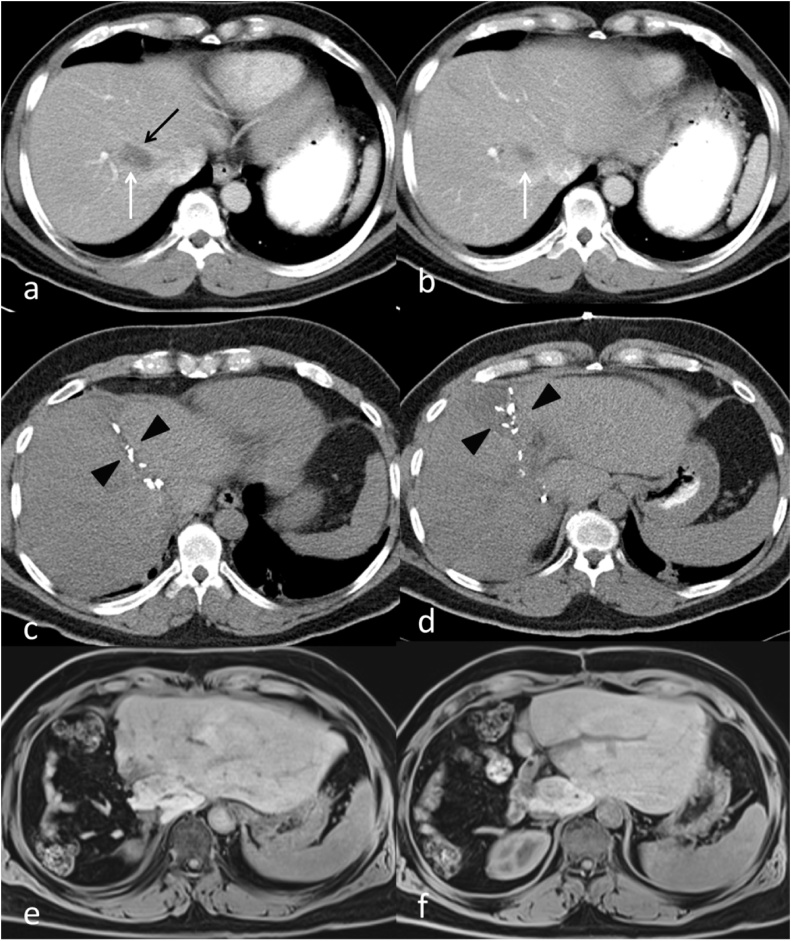
Patient 2: Preoperative contrast-enhanced CT (a,b) shows liver metastasis of the right lobe of the liver (white arrows), intimately related to the middle hepatic vein (black arrow). Post-stage-1 unenhanced CT on postoperative day 6 (c,d) shows the liver partition plane metallic clips (black arrowheads). Post-stage-2 unenhanced T1-weighted MRI at 6 months post stage 2 (e,f) shows obvious increase in size of the remaining liver.

**Fig. 5:**
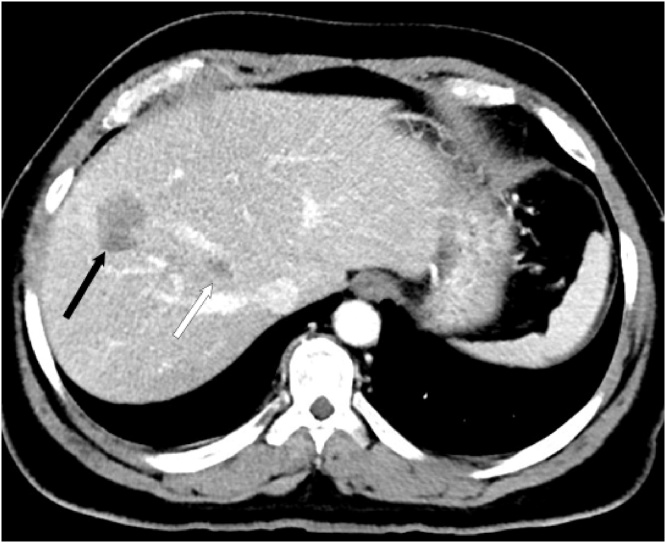
Patient 2: Preoperative contrast-enhanced CT shows new metastasis of the right lobe of the liver (Black arrow). The RFA treated old lesion is marked with a white arrow.

The second case was a 59-year old male patient with colorectal liver metastases. The patient was diagnosed with rectosigmoid cancer 18 months before his liver resection, which was T3 N2 M1 with a synchronous 8 mm liver metastasis in segment 8. The lesion was located in the angle between the middle and right hepatic veins at their junction with the Inferior Vena Cava (IVC), abutting them and making resection impossible without division of both veins (Fig. 4a and b). The liver was found to be markedly fatty on imaging that was mostly related to chemotherapy, and extended liver resection would be unsafe and would put the patient at risk of post-hepatectomy liver failure. He underwent laparoscopic anterior resection of the primary tumor and histopathology confirmed a pT3 pN2 lesion. Following recovery, he received 6 cycles of FOLFOX chemotherapy. He then underwent CT-guided Radio-Frequency-Ablation (RFA) ablation of the liver lesion with good response, followed by further 6 cycles of chemotherapy. Follow up images four months after the ablation showed a maintained complete response of the ablated lesion, but a new lesion was seen in segment 8 (Fig. 5). The lesion was again ablated by US guided RFA, with a good response after 4 weeks. 12 weeks post ablation; recurrent activity in the segment 8 lesion was noted on follow up MRI. A PET-CT scan confirmed uptake in the recurrent lesion, with no activity in the first ablated lesion, and showed no extrahepatic disease. The patient was planned for resection and it was decided that the new lesion in segment 8 should be resected together with the old lesion that responded to RFA because of concerns of pathological activity or recurrence despite the radiological response. An extended right hepatectomy was planned because the location of the first lesion between the middle and right hepatic veins mandated division of both veins to achieve an R0 resection. CT volumetry was done and the estimated FLR was 19.8% following an extended right hepatectomy, and ALPPS was planned due to the inadequate FLR.

**Fig. 6:**
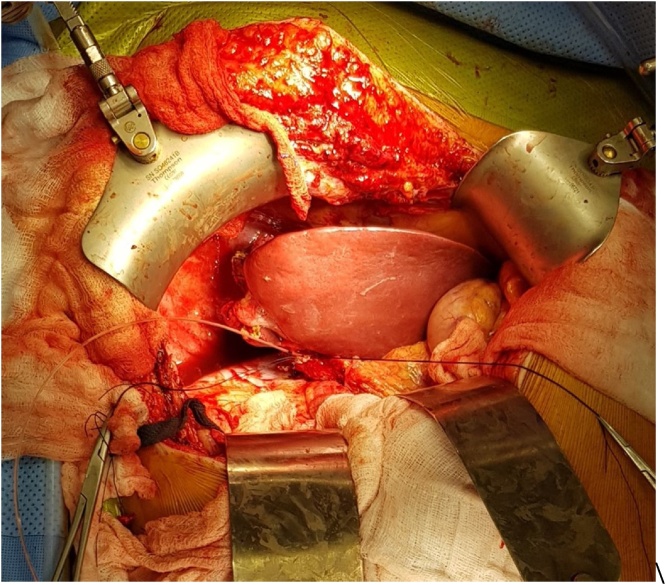
Patient 2:: Intraoperative photograph at the end of stage 2. A catheter is seen passing into the remnant of the right bile duct for a completion cholangiogram to confirm the anatomy and patency of the remaining biliary system.

At the first stage of ALPPS exploration showed no peritoneal disease. There were adhesions at the area close to the prior RFA between the middle and right hepatic veins, which were released, and the middle and right hepatic veins were dissected and each encircled with vascular loops. The right hepatic artery and bile duct were also identified, and each encircled with loose ties. The right portal vein was identified and divided with a vascular stapler. Parenchymal transection was done to the left of the middle hepatic vein in a modified extended right hepatectomy, and the dissection was carried down to the IVC. The right lobe was wrapped with a plastic sheet that also covered the resection margin, drains were placed, and the abdomen was closed. The procedure duration was 360 min with 500 ml of estimated blood loss. The patient had a smooth postoperative course. The liver enzymes and bilirubin peaked on postoperative day 1 to serum bilirubin of 24 mmol/l, ALT of 1118 U/L, and AST of 724 U/L but all dropped to normal levels at postoperative day 5. Follow up CT on postoperative day 6 demonstrated good hypertrophy of the FLR to 35.7% (Fig. 4c and d). This represented 80.2% increase in the FLR. The patient underwent the second stage next day (day 7), where the right and middle hepatic veins, the right hepatic artery, and the right bile duct were divided by stapler, and the diseased liver was removed. A healthy remnant liver with good hypertrophy was confirmed intra-operatively (Fig. 6). The patient had uneventful recovery after the second stage and was discharged home on postoperative day 4 in a good condition. After the second stage, bilirubin peaked again to 24 umol/L while the liver enzymes showed no significant increase. The bilirubin level decreased to normal on day 3 after the second stage. Histopathology showed the lesion in segment 8 to be necrotic with clear margins, while the newer lesion showed partially necrotic metastatic adenocarcinoma, with a 2 cm free margin. Last follow-up at 6 months after surgery revealed that the patient remained well with no complications and no evidence of tumor recurrence on imaging (Fig. 4e and f).

## 4 Discussion

In this report we describe the first 2 cases of ALPPS in Qatar that were done successfully with excellent outcome. The procedures were performed for sarcoma and colo-rectal liver metastases, and the indication for ALPPS was a low calculated FLR in both cases.

Resection of liver lesions, both metastatic and primary tumors, is the best, and usually the only, curative treatment. Obtaining clear margins in an R0 resection is the goal of resection. But, despite the improvement in resection techniques and safety of surgery, the main problem with extensive resection is the development of post hepatectomy liver failure that result when the remaining part of the liver, called the future liver remnant (FLR) is inadequate to maintain the normal liver function. This limits the extent of possible resection for bilateral or extensive tumors. This is compounded by the fact that most of the patients with liver tumors receive chemotherapy for extended periods prior to their resections, further compromising the liver function, with the need for a larger FLR to carry out normal function and minimize the risk of post-hepatectomy liver failure. The FLR is measured either as a percentage of volume of the FLR to the total liver volume before resection or as a percentage of the weight of the FLR to the patient’s weight. The minimum FLR to maintain function is more than 30% of the liver volume in a normal liver [[5], [6], [7]], but these limits increase to a FLR of more than 40% after chemotherapy or in pre-existing liver disease.

To enable resection of more volumes of the liver and thus increase the resectability of large tumors, techniques are used to increase the size of the FLR. The two classical approaches are portal vein embolization (PVE)/ligation (PVL) and two stage hepatectomy. ALPPS is a new modification. Two-stage hepatectomy involves portal vein ligation or embolization together with clearance of tumor deposits from the FLR. This is followed several weeks later by resection, after confirmation of the adequate FLR hypertrophy to an adequate volume. Portal vein ligation / embolization also help in increasing the volume of the FLR. The mechanism of increase in the FLR in both cases is believed to be the diversion of the portal blood to the FLR, and this induces both, hyperplasia and hypertrophy of the hepatocytes. The problem with these two procedures is the long time needed for hypertrophy (at least 6 weeks), during which tumor progression occurs in about 40% of the patients, eliminating the chance for resection. This problem is thought to be due to the opening up of portal shunts and collaterals within the liver parenchyma, thus decreasing the portal diversion.

ALPPS is a relatively new technique that helps to overcome this problem. In ALPPS, the occlusion of the portal vein is associated with division of the liver parenchyma along the line of future transection, while preserving the arterial supply. This results in accelerated growth of the FLR to the desired volume and completion of the second stage much faster, with most of the patients achieving the desired FLR volume within a median of 6–14 days in most patients (sometimes delayed to up to 30 days) [[8], [9], [10], [11], [12], [13]]. In the meantime, the diseased liver to be resected provides partial function that helps to maintain liver function until an adequate FLR is achieved. This allows almost 100% of the patients to proceed to the second stage and 86–100% have R0 resection [8,9,[14], [15], [16], [17]].

ALPPS was first performed in 2007 in Germany in one patient by chance, when the tumor planned for resection was found to be extensive intraoperatively. The surgeons have done partial resection of the liver before aborting then opted to ligate the portal vein on the side of the tumor. The patient was noted postoperatively to have a rapid increase in the size of the FLR [8,18]. The next description of the procedure was a poster presentation in the European – African HPBA meeting in 2007 by another group from Germany [8,18]and this was followed by the first published series of 25 patients in 2012 b [11]. Since that time, ALPPS evolved rapidly with variable adoption due to the highly reported morbidity and mortality figures.

ALPPS is indicated in patients with unresectable liver tumors due to a marginally adequate or inadequate FLR and when portal vein embolization would not be adequate. This includes all the patients with an inadequate FLR in addition to one of the following: “a tumor margin close to the FLR or its vascular pedicles, bi-lobar disease with contraindication for PVE, failure of PVE/PVL, unexpected tumor extension during surgical exploration with a larger than planned surgical resection, or the need for a large hypertrophy (>65%) in an extremely small FLR” [10]. ALPPS is contraindicated when there are unresectable lesions in the FLR or unresectable extrahepatic metastases, in severe portal hypertension, and in high surgical risk patients.

The standard description of ALPPS [11] involves right hepatectomy or right tri-sectionectomy in two stages. In the first stage, the liver is completely mobilized, and the parenchyma is divided along the planned transection line down to the IVC. The hepatoduodenal ligament is then dissected, and the right portal vein is ligated and divided, while preserving the hepatic artery and the bile duct. The right lobe of the liver is put in a plastic bag to prevent adhesions and the abdomen is closed. The patient is then followed with CT volumetry at day 7, and this is repeated weekly for 4 weeks until the required FLR is achieved [19]. When sufficient FLR hypertrophy is achieved, the patient is then taken for the second stage, where the right bile duct and the hepatic artery are both divided, the middle and right hepatic veins are ligated and divided, and the resection of the right lobe is completed [8,11]. Subsequent descriptions advised against the use of the plastic bag as it is associated with increased risk of infection.

Several variations of the original technique have been described to overcome the high incidence of septic complications, morbidity and mortality associated with the procedure. In Hybrid ALPPS, the liver is not completely mobilized in the first stage, transection is carried through an anterior approach and portal vein embolization is done radiologically postoperatively. The main objective is to decrease manipulation of the liver to improve the oncological outcomes. However, there is no proof of improved oncological outcome and the anterior approach is hazardous due to less vascular control. Bile duct ligation was also suggested by some authors, but it was abandoned due to the higher risk of bile leak and septic complications. Hernandez and colleagues [9] advocated decreased dissection of the hepatoduodenal ligament to decrease the possible ischemia to segment 4 and therefore decrease the incidence of bile leak and septic complications. Preservation of the middle hepatic vein is also described by Hernandez and it helps to decrease the venous congestion and septic complications. In ALTPS (Associating Liver Tourniquet and Portal vein occlusion for staged hepatectomy) a liver tourniquet is used instead of transection, and it produces hypertrophy of the FLR similar to that achieved with ALPPS, ranging from 33% to 189% [20]. Other modifications include using radiofrequency ablation instead of parenchymal transection in the first stage (Radiofrequency assisted liver partition, RALPP) [21], laparoscopic ALPPS, and the segmental variations. These segmental variations including left sided ALPPS, segment 4 ALPPS and mono segment ALPPS [19,22]

The increased hypertrophy in ALPPS is thought to be due to the interruption of the collaterals that develop from the lobe receiving the portal diversion to the de-vascularized lobe, thus creating a more complete diversion. This hypertrophy is mediated by two mechanisms: first is the humoral and growth factors released, and second is the stimulation by the increased portal flow. Failure of hypertrophy is sometimes seen and may be due to excessive portal pressure in the remaining FLR, in a condition similar to “small for size syndrome”. The other reason may be the effect of chemotherapy [8].

The common outcomes in the literature are the 90-day mortality, the FLR degree of hypertrophy, the number of patients proceeding to stage II, the rate of R0 resection, and the complications rate. The long-term outcomes are not commonly reported as the procedure is relatively new. The outcomes in published ALPPS reports are variable due to the evolving nature of the procedure, the different variations in the technique, and the heterogeneous patient population. In the initial published series, Schnitzbauer and colleagues [11] described a mortality of 14% at 6 months, with a median increase in the FLR of 74% (21–192%). Recent reports included more homogenous patient populations. Schadde and colleagues reported the outcome from 202 patients from the ALPPS registry with 141 patients with colo-rectal liver metastases. The median starting liver FLR was 21% and increased by a median of 80% in 7 days. The ninety-day mortality was 9%. Severe complications, defined Clavien-Dindo Grade IIIb or more, occurred in 26%.

The efforts to decrease the morbidity and mortality associated with ALPPS can be summarized in three directions: First, improving the selection of patients and defining the risk factors; second, decreasing the invasiveness of the first stage to decrease the septic complications; and third, improving the assessment of the FLR function before the second stage

Regarding the selection of patients, many risk factors have been associated with increased morbidity and mortality from ALPPS including age, surgery for biliary malignancies, increased operating time in the first stage, blood loss, and blood transfusion. These risk factors should be avoided or minimized [23]. In an effort to quantify the risk and improve selection, the group from Zurich University analyzed the data for 528 ALPPS patients and identified the risk factors associated with a futile outcome after ALPPS. They defined the futile outcome as 90 day or in hospital mortality and classified the risk factors according to the stage of surgery. Pre-stage I risk factors were found to be age >67 and biliary tumors, and pre- stage 2 risk factors were serum bilirubin, creatinine, pre-stage I score, and major complications. Through logistic regression, and using the regression coefficients, they devised an “ALPPS Risk Score” with two separate scores for the pre-stage I and pre-stage II. The risk for futile outcome for a pre-stage I score of 1,2,3,4 and 5 was: 2.7%, 4.8%, 8.6%, 15%, 24%, and 37% respectively. For the pre-stage II the risk for futile outcome was 5% for a score of 3.9, 10% for a score of 4.7, 20% for a score of 5.5, and 50% for a score of 6.9. This score helps to predict the risk and improve patient selection. Patients with high Pre-Stage-1 risk may not be offered the procedure and those with higher risk after stage I may not proceed to stage II.

A Less invasive approach described by Linecker and colleagues is partial ALPPS, with transection of only part of the parenchyma along the resection line. It is comparable to complete ALPPS in the level of hypertrophy that results. They compared cases of partial ALPPS (34–86% transection, with median of 61%), to complete ALPPS and to another external series with complete ALPPS. They concluded that there is no significant difference in hypertrophy if at least 50% transection was done, and that a partial ALPPS can provide a less invasive option [24]. However, experience shows that incomplete transection of the parenchyma makes the second stage extremely difficult with a high risk of bleeding.

Tanaka et al suggested that the increased morbidity and mortality with ALPPS is due to ischemic segments that result after division of the portal branches along the transection line. These ischemic segments act as a nidus for infection and increase the risk of sepsis, which is the main reason for mortality. They proposed a modified ALPPs procedure in which the portal pedicles along the transection lines are preserved to prevent ischemic areas. They have described successful outcomes with hypertrophy rates comparable to conventional ALPPS of 1.638 ± 0.384 a week after the first-stage procedure [25].

The third direction is to improve the assessment of the FLR function before the second stage by hepatobiliary scintigraphy. Volume assessment is just a surrogate measure, but the main question is whether the FLR has sufficient function that reflects the increase in volume. This is underlined by a recent review of the ALPPS registry where most of the mortality after stage 2 was due to post-hepatectomy liver failure, despite adequate FLR volume increase [26]. These patients should not have undergone the second stage if the function was properly assessed. After demonstrating that the volume and functional changes in the FLR after liver resection are similar in humans and in rabbit models [27], Olthof and colleagues subsequently demonstrated that the initial increase in volume is not associated with a similar increase in function of the FLR in these rabbit models [28]. The function was traditionally measured by conventional planar 99mTc-mebrofenin scintigraphy. But the FLR calculation is not accurate due to the anatomical position of the liver that results in smaller volume of the right hemi-liver on anterior and posterior projections [29]. A major advance in this field is the use of Single Photon Emission Computed Tomography (SPECT) combined with low dose CT scanning [29] which allows the accurate calculation of the FLR function, both as a percentage of the remaining liver and as an absolute function. Serenari and colleagues recently described the Hospital Italiano de Buenos Aires (HIBA) index derived from SPECT/CT and conventional hepatobiliary scintigraphy and were able to describe a cutoff value of 15% to predict the risk of post-hepatectomy liver failure, where 80% of the patients with an index < 15% developed liver failure, while none of the patients with an index >15% had liver failure. This remains an active area of development and new advances are expected to greatly reduce post-hepatectomy liver failure and consequently the mortality associated with ALPPS.

## 5 Conclusion

ALPPS is a relatively new technique that allows large liver resections that stretch the boundaries of the current limits and definitions of an adequate FLR. Refinements in the technique together with good patient selection help in establishing ALPPS as a safe option when large liver resections are needed.

## Please state any conflicts of interest

No conflicts of interest.

## Please state any sources of funding for your research

This research did not receive any specific grant from funding agencies in the public, commercial, or not-for-profit sectors.

## Ethical approval

The case report was approved by the Medical Research Centre of Hamad Medical Corporation (ABHATH) on March 20, 2018.

Protocol ID MRC-04-18-107.

## Consent

Written informed consents were obtained from the patients and are ready for review.

## Author contribution

Ibnouf Sulieman: Main author. Contributed to the writing of the case report, discussion and literature review, obtaining the required consents and ethical approvals, and the writing up of the manuscript.

Walid Elmoghazy: Surgeon: Conception of the idea of the case report, surgical care of the patients, and review and editing of the manuscript.

Mohammed Said Ghali: Contributed to the writing of the manuscript.

Ahmed Elaffandi: Surgeon: Reviewed and edited the manuscript.

Hatem Khalaf: Surgeon: Guidance and overall responsibility of the study; review and final approval of the manuscript.

Ahmed Mahfouz: Senior radiologist: Had input in the diagnosis of both cases and provided the images for publication with the comments.

## Registration of research studies

Research Registry UIN: researchregistry4672.

## Guarantor

Dr. Walid Elmoghazy.

Dr. Ibnouf Sulieman.
